# Surgical Approaches to Chronic Pancreatitis

**DOI:** 10.1155/2015/503109

**Published:** 2015-11-22

**Authors:** Daniel Hartmann, Helmut Friess

**Affiliations:** Department of Surgery, Klinikum Rechts der Isar, Technische Universität München, Ismaninger Straße 22, 81675 Munich, Germany

## Abstract

Chronic pancreatitis is a progressive inflammatory disease resulting in permanent structural damage of the pancreas. It is mainly characterized by recurring epigastric pain and pancreatic insufficiency. In addition, progression of the disease might lead to additional complications, such as pseudocyst formation or development of pancreatic cancer. The medical and surgical treatment of chronic pancreatitis has changed significantly in the past decades. With regard to surgical management, pancreatic head resection has been shown to be a mainstay in the treatment of severe chronic pancreatitis because the pancreatic head mass is known to trigger the chronic inflammatory process. Over the years, organ-preserving procedures, such as the duodenum-preserving pancreatic head resection and the pylorus-preserving Whipple, have become the surgical standard and have led to major improvements in pain relief, preservation of pancreatic function, and quality of life of patients.

## 1. Introduction

Chronic pancreatitis (CP) is an inflammatory condition characterized by a progressive loss of pancreatic parenchymal tissue that results in fibrosis, inflammation, and loss of exocrine and endocrine function [1]. It causes irreversible destruction of pancreatic parenchyma and leads to constant or recurring epigastric pain radiating to the back [2]. Alcohol abuse, pancreatic duct obstruction, systemic diseases, mutations of the cystic fibrosis gene, and hereditary or autoimmune pancreatitis have been found to be the main causes of CP. Progression of the disease can lead to complications such as pancreatic cancer development, duodenum and bile duct obstruction, or pseudocyst formation. Thus, medical and surgical treatment of CP aims to improve patients' quality of life by providing pain relief, improving pancreatic insufficiency, and reducing the number of complications [2].

With regard to pain in CP, several studies have shown a significant correlation between the severity of abdominal pain and the extent of intrapancreatic neural damage [3, 4]. Even though the pathophysiological mechanisms of neuropathic pain in CP have not yet been fully deciphered, recent studies point to a process of neural inflammatory cell infiltration leading to pancreatic neuritis and neural plasticity resulting in the formation of a dense intrapancreatic neural network [2, 5, 6]. Pain management strategies include the intake of pancreatic enzyme supplements, cessation of alcohol intake and smoking, and consequent analgesic therapy. In case of persistence of symptoms or suspicion of pancreatic cancer, surgical procedures come into play.

## 2. Surgical Techniques for Painful CP

Since the early 20th century, surgical treatment has been applied in CP patients who fail medical therapy, initially making use of pancreatic duct drainage by pancreatostomy [7]. In the ensuing decades, Puestow and Gillesby developed a technique that combined a longitudinal opening of the pancreatic duct and an anastomosis to the small intestine with a pancreatic left resection [8]. Shortly thereafter, Partington and Rochelle modified this procedure with an extended opening of the pancreatic duct and a preservation of the pancreatic tail, and their version is still being used nowadays for dilated pancreatic ducts of more than 7 mm in size [9]. As pointed out by these attempts of surgical treatment of CP, drainage operations were considered first; however, it has been shown that only a few procedures carry good long-term results [10]. Thus, several surgical procedures for painful CP that have been developed thereafter aimed at the resection of the pancreatic head carrying an inflammatory mass, such as (i) the standard Kausch-Whipple procedure with resection of the complete pancreatic head, gallbladder, duodenum, and gastric antrum, (ii) the pylorus-preserving pancreaticoduodenectomy (Traverso-Longmire procedure), or (iii) the total pancreatectomy in severe cases of CP with affection of the entire gland [2]. The Beger procedure, a resection of the pancreatic head that preserves the duodenum and intrapancreatic bile duct by subtotally excising the head and uncinate process, was developed in 1972 [11]. One decade later, the Frey procedure offered a less invasive organ-preserving variation of the Beger procedure that combined longitudinal pancreaticojejunostomy with a local pancreatic head excision without transection of the pancreas above the portal vein [12]. Further modifications of duodenum-preserving pancreatic resections exist, such as the Berne/Farkas technique developed independently by Büchler and colleagues and Farkas and colleagues, which consists of a partial resection of the pancreatic head without a lateral pancreaticojejunostomy [13]. This operation avoids the transection of the pancreas above the portal vein and combines the advantages of the Beger and Frey operations.

Additional surgical procedures have been subsequently developed to resolve CP-related complications but also to preserve pancreatic parenchyma as much as possible. Kutup and his team developed the longitudinal “V-shape excision” of the ventral pancreas in patients suffering from “small duct” pancreatitis (pancreatic duct diameter < 3 mm) [14]. In patients with a focal CP in the corpus of the pancreas, a middle segmental pancreatic resection has been developed as another organ-preserving operation [15]. Depending on pathology and location, other procedures include pancreatic left resection or total pancreatectomy [16].

Please see Table 1 for an overview of different surgical techniques for the treatment of CP.

## 3. Randomized Controlled Trials on the Endoscopic and Surgical Treatment of CP

One major controversial issue about the treatment of painful obstructive CP is whether surgical therapy is superior to endotherapy. The argument of interventional endoscopists is that surgery is too invasive and CP-related problems can be successfully treated by stenting. In order to analyze this issue, Díte et al. performed the first prospective, randomized controlled trial comparing surgery with endoscopy in a total of 72 patients with painful CP caused by ductal obstruction in 2003 [21]. Within the surgery arm, 20% of cases received a drainage procedure and 80% had a resection performed; within the endotherapy arm, 52% of patients underwent a sphincterotomy and stenting; 23% a sphincterotomy, stenting, and stone removal; and 23% solely a stone removal [21]. Initial pain relief success rates were above 90% in both groups at one year, but these results changed significantly after 3 and 5 years [21]. At the 5-year follow-up, surgery was found to be superior to endotherapy in both the whole group and the randomized subgroup for long-term pain reduction and increase in body weight [21].

A few years later, Cahen et al. performed a similar randomized trial on 39 symptomatic CP patients with a distal obstruction of the pancreatic duct in order to compare endoscopic and surgical ductal decompression either by endoscopic transampullary drainage (19 patients) or operative pancreaticojejunostomy (20 patients) [22]. After the first unscheduled interim analysis, the study had to be terminated because of ethical considerations (a decision of the safety committee based on a significant difference in outcome in favor of the surgical group). Compared with the stenting group, significantly better pain and physical health scores were found for CP patients undergoing surgical drainage of the pancreatic duct (*p* values < 0.001 and 0.003, resp.), while rates of complications, length of hospital stay, and changes in pancreatic function were similar within the two treatment groups [22]. In 2011, the authors published results on the long-term outcomes of these patients after another 5-year follow-up and were able to show that 47% of patients who underwent endoscopic transampullary drainage did need a surgical intervention in the end [23]. In addition, patients assigned to the surgical treatment arm needed significantly fewer procedures than endoscopically treated CP patients (4 versus 12; *p* value = 0.001) and had more relief from pain (80% versus 38%; *p* value = 0.042), while levels of quality of life and pancreatic function were similar [23]. The only drawback within this context is the fact that the study by Cahen et al. was not sufficiently powered for long-term follow-up analysis. Thus, the data from 2011 show lower significance levels compared to the original study from 2007 with a follow-up period of 24 months because there were fewer cases to consider over the long run. All in all, these randomized controlled trials give strong evidence that surgical therapy provides significantly better long-term results than endoscopic interventions.

Please see Table 2 for an overview of randomized controlled studies comparing endoscopic and surgical treatment of painful CP.

Since worldwide consensus and standard criteria on the choice of surgical procedure are lacking, the work of Cahen and colleagues has gained importance. Patients with a dilated pancreatic duct in the absence of an inflammatory mass in the pancreatic head can be effectively treated with an operative pancreaticojejunostomy [22, 24]. Different options exist with regard to the inflammatory pancreatic head mass, which is thought of as being the pacemaker of pain and progression of disease [24]. Thus, in patients with an enlarged pancreatic head, appropriate surgical options are pancreatoduodenectomy or duodenum-preserving pancreatic head resection, as mentioned above [24]. Radical pancreatic resectional procedures are also being carried out in the case of small duct CP with a nondilated main pancreatic duct and an associated head mass of uncertain etiology [25]. In particular, with regard to small duct CP with head dominant disease, several randomized trials have shown that duodenum-preserving pancreatic head resection and its modifications provide excellent long-term pain relief and can be considered the standard for this form of CP [25].

An important and oftentimes underestimated aspect is the timing of surgery in the management of patients with CP: early or late in the course of the disease. A recent systematic review and meta-analysis supports early surgery for pain management in CP patients because early surgical intervention is associated with improved postoperative pain relief, reduced risk of pancreatic insufficiency, and decreased reintervention rates in comparison with conservative step-up approaches [26]. A similar observation was made in the study by Cahen et al. that analyzed long-term follow-up data. Almost half of the patients who were initially treated endoscopically underwent salvage surgery. However, delayed surgery was not as effective as expected, indicating that postponing surgery in CP patients has a negative influence on the treatment outcome [23].

## 4. Randomized Controlled Studies on the Surgical Treatment of CP

Evidence-based data on the surgical treatment of CP favors tailored organ-sparing procedures, such as the Beger or Frey procedures, over the classic Whipple or the pylorus-preserving Whipple procedure. In two monocentric randomized controlled trials, Klempa et al. and Büchler et al. found duodenum-preserving pancreatic head resection to be superior to the classical Whipple procedure in regard to pain relief, weight gain, and endocrine pancreatic function [13, 27]. Comparing 21 patients treated with the classic Whipple and 22 patients treated with the Beger procedure, Klempa et al. also found the duodenum-preserving pancreatic head resection to lead to a significantly shorter hospital stay [27]. After long-term follow-up of the randomized clinical trial (14 years) from Heidelberg, these advantages were no longer significant [28]. However, it should be stated in this context that the original study had not been powered for long-term follow-up, and significance levels decreased because patients were lost to follow-up or passed away. There is a statistical limit in all studies listed in Table 3, because none of these studies was powered for long-term follow-up investigations. Therefore, there is a clear tendency for a better outcome following organ-preserving surgery compared to the Whipple operations.

In addition, two independent randomized trials by Farkas et al. and Izbicki et al. revealed that the Frey procedure provides a better quality of life, while the pylorus-preserving pancreaticoduodenectomy (pp Whipple) and the Frey procedure were found equally effective in pain relief [29, 30]. Long-term follow-up showed comparable pain control and pancreatic function between both procedures, while survival rates were superior after the Frey procedure [31, 32].

In a prospective, randomized trial on different techniques of duodenum-preserving pancreatic head resection, Izbicki et al. compared the Beger and the Frey procedures and found both equally safe and effective in pain relief, postoperative quality of life, control of complications affecting adjacent organs, and exocrine or endocrine pancreatic function [33]. About ten years later, Strate et al. published a long-term follow-up of these data showing no difference in mortality, quality of life, pain, or exocrine or endocrine insufficiency within the two groups [34]. In addition, a controlled, prospective, randomized study by Köninger et al. on the evaluation of the Beger and Berne/Farkas procedures for CP showed that the Berne/Farkas technique provided significantly shorter operation times and hospital stays, while the quality of life was found to be similar [35]. In a recent randomized trial on pylorus-preserving and duodenum-preserving pancreatic head resections, Keck et al. found both types of resections to be equally effective in pain relief and quality of life without differences in exocrine or endocrine pancreatic function [36].

Taken together, the duodenum-preserving pancreatic head resections (including Beger, Frey, and Berne/Farkas procedures) have been shown to be superior to the classic Whipple procedure, while Frey and Beger procedures have similar results when compared to each other.

Please see Table 3 for an overview of randomized controlled studies on the surgical treatment of painful CP.

A systematic review and meta-analysis of four randomized controlled trials on the surgical treatment of pancreatic head lesions in CP patients by Diener et al. showed duodenum-preserving pancreatic head resections (including Beger, Frey, and Berne/Farkas procedures) to be superior in peri- and postoperative outcome parameters and quality of life compared to partial pancreatoduodenectomy (Whipple procedure) while being equally effective in terms of postoperative pain relief, overall health, and postoperative endocrine sufficiency [38]. Another meta-analysis including 15 studies by Yin et al. revealed that pancreaticoduodenectomy offered significantly less postoperative pain relief than the Beger procedure and worse postoperative morbidity than the Frey procedure, while quality of life, pancreatic exocrine function, and delayed gastric emptying also favored duodenum-preserving strategies [39].

Another procedure that has been evaluated lately as a treatment option for a subset of CP patients is the method of total pancreatectomy and autologous islet transplantation (TP/IAT).

In a systematic review of the literature, TP/IAT has been shown to successfully reduce pain, while a significant proportion of patients are able to remain independent of insulin supplementation even in the long run [40]. Nevertheless, the impact of this surgical procedure on quality of life and its optimal timing in relation to the evolution of CP has not been studied sufficiently [40]. Therefore, it should only be considered as ultima ratio once all other surgical treatment options have been exhausted or are unlikely to improve the symptoms of these patients [40].

An additional example of problem-tailored surgery is portal hypertension. In most cases, surgery does provide good relief of portal hypertension [41]. Splenectomy and gastric devascularization are considered adequate treatment options for CP patients with bleeding gastric varices due to portal hypertension [41]. Several new developments have been made with respect to left-sided portal hypertension, which was considered a relative contraindication to laparoscopic splenectomy. In a recent case study, Patrono and colleagues reported on the management of a CP-related splenic vein thrombosis by laparoscopic splenectomy after splenic artery embolization [42].

## 5. Conclusion

With reference to postoperative functional outcome after surgical treatment of painful CP, a deterioration in pancreatic function can be seen in most cases, which is most likely inevitable after removal of pancreatic tissue [2]. In any case, surgery should be tailored to the needs of patients and should be as problem-oriented and organ-sparing as possible. Therefore, based on present evidence, patients suffering from CP and its associated complications (e.g., pain, duodenal or pancreatic duct obstruction, cholestatic jaundice, appearance of an inflammatory mass, and portal hypertension) should be evaluated for problem-tailored surgery in an interdisciplinary center with expertise in pancreatic surgery.

Unfortunately, many patients are sent for surgery at a prolonged disease stage, when even surgery cannot be an effective treatment anymore. Nowadays, surgical treatment of CP is associated with low morbidity and mortality, preservation of exocrine or endocrine pancreatic function, sustainable pain reduction, and major improvement in quality of life.

In particular against this background, one should bear in mind that an organ-sparing operation at the right point in time carries a better outcome than performing an extended resection, like a Whipple operation, as a last resort once all therapeutic options are exhausted. For this reason, early timing of surgical therapy is crucial for the outcome of patients with painful CP, and the indication of surgery should be considered early once symptoms are unambiguous.

## Figures and Tables

**Table 1:** Surgical techniques for the treatment of CP.

Authors	Publication	Surgical procedure
Link	Ann. Surg. 1911 [7]	Pancreaticojejunostomy
Kausch	Beitr. z. klin. Chir. 1912 [17]	Pancreaticoduodenectomy
Whipple et al.	Ann. Surg. 1935 [18]	Pancreaticoduodenectomy
Partington and Rochelle	Ann. Surg. 1960 [9]	Pancreaticojejunostomy
Puestow and Gillesby	AMA Arch. Surg. 1958 [8]	Pancreaticojejunostomy
Beger et al.	Acta Chir. Scand. 1990 [11]	Duodenum-preserving pancreatic head resection
Traverso and Longmire	Surg. Gynecol. Obstet. 1978 [19]	Pylorus-preserving pancreaticoduodenectomy
Frey and Smith	Pancreas 1987 [12]	Duodenum-preserving pancreatic head resection (Frey operation)
Büchler et al.	Am. J. Surg. 1995 [13]	Duodenum-preserving pancreatic head resection (Bern procedure)
Izbicki et al.	Ann. Surg. 1998 [20]	Pancreaticojejunostomy (V-shape)

**Table 2:** Randomized controlled studies comparing endoscopic and surgical treatment of painful CP.

Authors	Publication	Number of patients	Results
Díte et al.	Endoscopy. 2003 [21]	72	Surgery better than endoscopy
Cahen et al.	N. Engl. J. Med. 2007 [22]	39	Surgery better than endoscopy
Cahen et al.	Gastroenterology. 2011 [23]	39	Surgery better than endoscopy

**Table 3:** Randomized controlled studies on the surgical treatment of painful CP.

Authors	Publication	Number of patients	Results
Izbicki et al.	Ann. Surg. 1995 [33]	42	Frey equal to Beger
Izbicki et al.	Chirurg 1997 [37]	74	Frey equal to Beger
Strate et al.	Ann. Surg. 2005 [34]	74	Frey equal to Beger
Klempa et al.	Chirurg 1995 [27]	43	Beger better than Whipple
Büchler et al.	Am. J. Surg. 1995 [13]	40	Beger better than Whipple
Köninger et al.	Surgery 2008 [35]	65	Berne/Farkas better than Beger
Izbicki et al.	Ann. Surg. 1998 [30]	61	Frey better than Whipple
Farkas et al.	Langenbecks Arch Surg. 2006 [29]	40	Frey better than Whipple
Bachmann et al.	Ann. Surg. 2013 [31]	64	Frey better than Whipple
